# Virus Dynamics and Evolution: Bridging Scales and Disciplines

**DOI:** 10.3390/v3081432

**Published:** 2011-08-16

**Authors:** Mary Poss

**Affiliations:** 1 Department of Biology, Center for Infectious Disease Dynamics, The Pennsylvania State University, University Park, PA 16802, USA; E-Mail: mposs@bx.psu.edu; Tel.: +1-814 867-1213; Fax: +1-814 865 9131; 2 Fogarty International Center, National Institutes of Health, Bethesda, MD 20892, USA

## 1. Introduction

Viruses have attracted the interest of researchers from multiple disciplines and have nucleated many productive and innovative collaborations. In part, this is because viruses so intimately associate with their hosts that decoupling host and virus biology is difficult, and virus-host interactions occur at multiple scales, from within cells to populations, each of which is intrinsically complex. As a consequence, ecologists, population biologists, evolutionary biologists, and researchers from quantitative fields, including mathematics, statistics, physics and computer science, make significant contributions to the field of virology. Our understanding of virus dynamics and evolution has substantially benefited from these multidisciplinary efforts. It is now common to see advanced phylogenetic reconstruction methods used to determine the origins of emergent viruses, to estimate the effect of natural selection on virus populations, and to assess virus population dynamics. Mathematical and statistical models that elucidate complex virus and host interactions in time and space at the molecular and population level are appearing more regularly in virology and biomedical journals. Massive quantities of data now available due to technological innovation in imaging, increased disease surveillance efforts, and novel approaches to determine social contact structure are changing approaches to study the dynamics and evolution of viral infections in heterogeneous environments. The next decade presents exciting new opportunities and challenges for the expanding field of researchers investigating dynamics of viral infections that will lead to innovation and new insight on virus interactions in both individual hosts and in populations. The compilation of articles in this *Special Issue* on “Virus Dynamics and Evolution” is comprised of reviews and primary research, summarized below, that provide new perspectives on virus interactions with host organisms through the integration of empirical and computational analyses of virus at molecular, cellular, and population levels.

### 1.1. Virus Dynamics and Evolution within Hosts

Tissue culture is a mainstay for virologists studying cellular response to virus infection and efficacy of therapeutic interventions and treatment; response variables are typically aggregate counts of infected cells or production of a soluble factor. However, averaging values over cells, which are in different growth phases or stages of viral infection, can obscure or confound important responses to virus infection. Simenov *et al.* [1] report on statistical approaches to determine the effect of heterogeneity of cells in culture, which increase resolution of response parameters in *in vitro* infection experiments. These investigators assess the dynamics of host innate defenses to two strains of Respiratory Syncytial Virus (RSV) that have distinct epidemiological patterns but can coinfect an individual. The authors used spatial statistical methods to evaluate how the resistance of naïve cells to a challenge infection with one of the RSV strains changed as a function of proximity to a cell infected with a different RSV strain and the time between the primary and challenge infection. In this experimental setting, every infected cell is a data point. An important result of their study is that even in cell lines, asynchrony in growth and replication in a culture can change the susceptibility of cells to infection, which is presumably due to an intrinsic property of replicating or differentiating cells and not an innate immune response to virus infection. However, despite the background heterogeneity in cell susceptibility to infection, the authors demonstrate that one RSV strain, but not the other, can limit the ability of the heterologous strain to establish infection; an effect that would be obscured if the authors had used aggregate instead of point process analysis in their study. Further development of spatial statistical models will be imperative as data on responses of individual cells in population of cells become more widely available.

Three manuscripts in this *Special Issue* on “Virus Dynamics and Evolution” reveal important features of virus-host interactions by integrating detailed knowledge of viral life history strategy with viral sequence evolution over the course of infection of the natural host organisms. Virus evolutionary changes are determined by mutation rate and viral generation time and by selective pressures imposed by the host environment. These metrics inform variation in virus replication under changing host conditions. However, there are many features of virus life history strategy that can confound estimating these standard evolutionary parameters. For example, the efficacy of viral strategies to modulate host immunity is not constant over time. As the host environment changes, so does viral replication and this can complicate rate estimates that are based on viral sequence data. Harrison *et al.* [2] explain that rate estimates for Hepatitis B Virus (HBV) are too fast to account for the long history shared by HBV and humans and too slow to account for the level of global HBV diversity. They demonstrate that rates vary dependent on expression of HBV e antigen (HBeAg). HBeAg is produced early in infection and damps the host immune response to viral core antigen. Mutations in HBeAg can prevent expression, which enhances immune pressure on the virus, and consequently alters virus evolutionary rate. Thus, the authors show that an understanding of the biology of HBV infection is key to deriving interpretable evolutionary parameters and that evolutionary models must consider rate differences for datasets that include both HBeAg positive and negative sequences.

Carpenter *et al.* [3] address another feature common to viruses that can confound interpretation of forces shaping virus evolution. Viruses increase the number of proteins that they encode by using overlapping reading frames. Because selection acts on more than one protein encoded by the same gene, the evolution of all proteins encoded by the gene should be constrained. However, the Rev protein of equine infectious anemia virus (EIAV), which has its second exon encoded in an alternative open reading frame with the viral envelope glycoprotein, is highly variable. Rev is essential for nuclear export of unspliced RNA produced in lentivirus infections but also contains CTL epitopes. Thus variation in Rev is on one hand constrained by encoding an essential function and because of sharing nucleotide sequence with another essential protein while simultaneously being driven to vary by host cellular immunity. EIAV causes cycles of viremia and thrombocytopenia interspersed between periods of viral control without disease. Carpenter *et al.* [3] discuss how two populations of Rev variants cycle in phase with disease symptoms. Substitutions distinguish each group and confer different nuclear export properties on Rev despite the fact that the substitutions occur outside the known nuclear export domain. This important result demonstrates that viruses can evolve multiple mechanisms to confer an essential function on a protein if there are putative constraints imposed by overlapping reading frames. Because Rev also controls the pace of viral gene expression and particle production, it is likely that variants that arise in persistent infection also exhibit different evolutionary rates, as discussed by Harrison *et al.* [2] for HBV.

Viral infections, although complex on their own, rarely occur in isolation. Polymicrobial infections can exacerbate clinical consequences to the host but can also attenuate disease. Feline immunodeficiency virus (FIV) infection of its domestic cat host is an exemplary system to explore the mechanisms by which an avirulent FIV strain (PLV) attenuates CD4 cell loss induced by a virulent FIV in the absence of measurable antibody or cellular defenses [4]. Padhi *et al.* [5] compared the viral population dynamics of FIV from PBMC in the presence and absence of PLV to determine if dual infection altered FIV population structure. The authors employed population genetics approaches because there was no evidence of FIV phylogenetic structure in either the presence or absence of apathogenic PLV over the three month experiment. Further, quantitative measures of virus census size did not reveal significant differences in FIV from single and dual infections at most time points [4]. However, FIV population dynamics were clearly impacted by PLV as evidenced by a lower effective population size during the early expansion phase of FIV infection and most notably by a bottleneck at approximately three weeks post-FIV infection. Several hypotheses were proposed to account for the impact of PLV on FIV population dynamics at multiple time points, which the authors summarize in a conceptual model. This study highlights the rich data inherent in evolving virus genome sequences and the value of using multiple analytical approaches to study virus interaction in a changing host environment.

### 1.2. Virus Evolution and Dynamics in Populations

The extensive genetic diversity in any virus population provides ample opportunity to improve viral fitness in a population of hosts. Theory suggests that viruses with more than one host, such as arboviruses, will be unable to specialize on either their vertebrate or insect hosts because of trade-offs affecting replication in either ([6] and references within). These constraints are proposed to explain why the evolutionary rates of arboviruses are slower than most error prone RNA viruses. Ciota *et al.* [6] review the experimental studies that have evaluated the evolution of important and recently emerged arboviruses in two-host environments. In both tissue culture experiments and *in vivo* infection, virus cycling among host species does not unambiguously constrain virus fitness in either host or lead to a fitness trade off if specialization occurs. On the contrary, fitness can increase in sequential passage of virus between insects and vertebrates and, of significance, virus fitness in some vertebrate or mosquito species may be less constrained than in related host species. Thus, specialization or enhanced fitness in one environment does not necessitate trade-offs. These experimental studies highlight important areas for future research. Much of the work in this field relied on consensus sequencing and therefore failed to assess the full extent of virus diversity. Advances in sequencing technology will allow thorough exploration of sequence diversity from *in vitro* evolution experiments. However, our understanding of the significance of diversity in viral populations is also limited (also discussed in Roossinck [11]). Detailed studies on are needed to determine how the diversity of the virus mutant spectrum affects disease, transmission, and host response.

Whereas virus life history strategy is a key factor driving virus dynamics and evolution within a host, host life history, behavior, and social structure have profound effects on the patterns of virus transmission and consequently the dynamics of viruses in populations. For example, the number and type of contacts an individual has affects virus transmission in a specified population. Network science provides an intriguing approach to study virus spread in a population of known contact structure [7]. The relatively new application of network analysis to epidemiology provides modeling alternatives for determining the size of an outbreak and the impact of interventions but they require that the contact structure, which is rarely available, is known [8]. The path an infection takes through a population, the transmission tree, is a subset of the contact network and can be inferred from both sequence data and epidemiological history of an infection in a population of interest. It is not known how differences in individual connectedness affect transmission dynamics but resolution of this problem could advance efforts to reconstruct contact networks from the transmission tree. Welch [9] investigates this question computationally by asking whether networks with the same overall number of contacts but different degrees of clustering can be inferred from virus transmission data. The results of simulations indicate that the spread of a virus in a highly clustered population is somewhat slower and more prolonged, and fewer overall infections occur at the outbreak peak. This pattern can be captured just using the transmission tree though statistical detection of the pattern may be difficult. The conclusions suggest that the overall number of contacts, which were held constant in the simulations, may have a stronger influence on epidemic parameters than clustering patterns. Integrating transmission and sequence data from viral infections with information on dynamics of host behavior that influence contact frequency is an exciting new area of research that will rapidly develop commensurate with increased data available from virus sequencing, disease surveillance, and social networking determination.

### 1.3. Ecological Impacts on Virus Dynamics and Evolution

Virus infections affect the population structure of host organisms and can have a profound influence on the interaction among individuals and their environment. Hence, the consequences of virus and other pathogen infection are central to the interests of ecologists. Vandegrift *et al.* [10] discuss the interrelation of virus infections and ecological processes and review the contributions of disease ecologists towards synthesizing virus dynamics in complex ecological systems. Emerging viral infections commonly originate from an animal reservoir but we know little about viral infections in wildlife, as evidenced by recent zoonotic emergence events from birds, bats, and rodents. New sequencing technology provides the potential to detect sequences of unknown viruses and to define the entire microbial population in an infected wildlife host or population; the microbial community can affect the risk of cross species transmission events by affecting host behavior and virus production. Virus transmission is an aspect of the viral life cycle that is not frequently studied by molecular virologists because mechanisms leading to infection and disease are a primary focus. However, at population levels, the rates and patterns of virus spread must be known to develop predictive models of epidemic risk and optimize intervention strategies. Similarly, the number of individuals and how they are distributed and interact within a population are needed to determine factors leading to a cross-species transmission event. Contemporary changes in the demographics of most wildlife species are difficult to determine using host genetic or individual marking methods. Because virus transmission events can be inferred based on virus sequences isolated from individuals in a population, virus genomes can be used as a proxy host gene to inform host population structure. Recently virus sequence data has been used to estimate population growth or decline, contemporary immigration events, and as discussed in Welch [9] in this issue, to estimate contact networks. The authors also present an important perspective on the effect of vaccination to host ecology and virus evolution. Vaccination affects transmission and has the potential to significantly change viral dynamics in a population. Vaccination can drive one virus to extinction but open a niche for other viruses to take hold. Intervention and surveillance efforts will be more efficacious if the ecology of the system in which infection risk is to be mitigated is known.

At cellular to population scales, our paradigms in virology are largely based on virus infections that have a detrimental affect on the host. However many—and probably most—viruses are not pathogens and some are beneficial to their host. Not surprisingly, the ecology and biology of apathogenic viruses have not been extensively investigated. The article by Roossinck [11] provides a welcomed perspective on the importance of mutualistic viruses and the needed research to better understand molecular and ecological consequences of mutualism. Viruses are not irreversibly committed to a particular life history strategy. Viruses that are virulent in one population may be apathogenic or mutualistic in other species or at a different time point in the same host. For example, our historical encounters with retroviruses are recorded in the primate genome as a consequence of the obligatory integration of this virus family into the genome of the infected cell; if that cell is an egg or sperm the virus effectively becomes a host gene and can be past vertically. Deleterious effects of germline integration are rapidly lost in a population, so contemporary examples of endogenous retroviruses demonstrate neutral and sometimes beneficial effects to the host. Whereas, the host dictates the evolution of endogenous retrovirus, polydnaviruses of parasitic wasps and fungal mutualist Curvularia Thermal Tolerance virus retain independent life cycles but confer a fitness advantage on their host. Pathogenic viruses often have high rates of RNA virus evolution and extensive population diversity but there is a lack of data on evolutionary dynamics in mutualistic and symbionic viruses, although available data suggest lower population diversity. Because rapid evolution complicates antiviral therapeutic and intervention strategies, understanding how RNA viruses with error-prone replicases can sustain such different levels of diversity and what constitutes the selection pressures that promote or constrain diversity would significantly enhance our mechanistic understanding of virus evolutionary strategies.

## 2. Conclusions

Virus infection is inherently a dynamic process from the protein to the population level. The articles in this *Special Issue* reveal new information about virus host interaction at each of these scales and demonstrate the unique perspective that virus evolution provides into the complex environment of the host. New tools will continue to capture virus dynamics and evolutionary change at finer spatial and temporal scales, which will lead to new challenges and opportunities for researchers trained in a variety of disciplines to contribute to data analysis and interpretation. Collectively, these efforts will advance our knowledge of viral pathogenesis, elucidate innovation in evolving protein structures, provide specific targets for therapeutic intervention, and inform models of infection spread in heterogeneous populations.
